# Spinal Muscular Atrophy: An Evolving Scenario through New Perspectives in Diagnosis and Advances in Therapies

**DOI:** 10.3390/ijms241914873

**Published:** 2023-10-03

**Authors:** Ilaria Angilletta, Rossella Ferrante, Roberta Giansante, Lucia Lombardi, Alessandra Babore, Anastasia Dell’Elice, Elisa Alessandrelli, Stefania Notarangelo, Marianna Ranaudo, Claudia Palmarini, Vincenzo De Laurenzi, Liborio Stuppia, Claudia Rossi

**Affiliations:** 1 Center for Advanced Studies and Technology (CAST), “G. d’Annunzio” University of Chieti-Pescara, 66100 Chieti, Italy; ilaria.angilletta@studenti.unich.it (I.A.); rossella.ferrante@unich.it (R.F.); roberta.giansante@studenti.unich.it (R.G.); lucia.lombardi@unich.it (L.L.); anastasia.dellelice@studenti.unich.it (A.D.); elisa.alessandrelli@studenti.unich.it (E.A.); stefania.notarangelo@studenti.unich.it (S.N.); marianna.ranaudo@studenti.unich.it (M.R.); palmariniclaudia@gmail.com (C.P.); vincenzo.delaurenzi@unich.it (V.D.L.); liborio.stuppia@unich.it (L.S.); 2 Department of Neurosciences, Imaging and Clinical Sciences, “G. d’Annunzio” University of Chieti-Pescara, 66100 Chieti, Italy; 3 Department of Psychological, Health and Territory Sciences, “G. d’Annunzio” University of Chieti-Pescara, 66100 Chieti, Italy; a.babore@unich.it; 4 Department of Innovative Technologies in Medicine and Dentistry, “G. d’Annunzio” University of Chieti-Pescara, 66100 Chieti, Italy

**Keywords:** spinal muscular atrophy, gene therapy, newborn screening, disease-modifying therapies, diagnosis, psychological adjustment

## Abstract

Spinal muscular atrophy (SMA) linked to 5q is a recessive motor neuron disease characterized by progressive and diffuse weakness and muscular atrophy. SMA is the most common neurodegenerative disease in childhood with an incidence of approximately 1 in 6000–10,000 live births, being long considered a leading cause of hereditary mortality in infancy, worldwide. The classification of SMA is based on the natural history of the disease, with a wide clinical spectrum of onset and severity. We are currently in a new therapeutic era, that, thanks to the widespread use of the newly approved disease-modifying therapies and the possibility of an early administration, should lead to a deep change in the clinical scenario and, thus, in the history of SMA. With the aim to achieve a new view of SMA, in this review we consider different aspects of this neuromuscular disease: the historical perspective, the clinical features, the diagnostic process, the psychological outcome, innovation in treatments and therapies, the possibility of an early identification of affected infants in the pre-symptomatic phase through newborn screening programs.

## 1. Introduction

Spinal muscular atrophy (SMA) linked to 5q is an autosomal recessive neuromuscular disease characterized by degeneration of alpha motor neurons in the anterior horns of the spinal cord, resulting in progressive and symmetrical proximal muscle weakness [1,2,3,4,5]. In particular, the main clinical manifestation of SMA includes hypotonia, muscle weakness by denervation, followed by respiratory failure and atrophy of variable severity depending on the genotype [6]. SMA has long been considered the leading cause of hereditary death in infancy worldwide [5,6], being second for incidence only to cystic fibrosis. The widespread use of the newly approved disease-modifying therapies (DMTs) and the possibility of an early administration should radically change the history of SMA [7]. The incidence of SMA is estimated at 1 in 6000–10,000, with a carrier frequency of 1/40–1/60 [8,9]. Nowadays, we know that most cases of SMA have mutations in the motor neuron type 1 (*SMN1*) gene located at 5q13.12. This gene covers 20 kb and is located in the telomeric portion of DNA that is subject to reorganization and deletion. The centromeric duplicated element, known as the *SMN2* gene, is highly homologous to *SMN1* with more than 99% nucleotide identity. *SMN1* and *SMN2* differ in eight nucleotides, specifically, the c.840C > T nucleotide change in *SMN2* is in a coding region and interrupts an exonic splicing enhancer in exon 7. Consequently, most *SMN2* transcripts lack exon 7, making a non-functional protein. Only approximately 10 percent of the proteins produced by each *SMN2* copy are estimated to be functional. SMN is an RNA-binding protein that contributes to many cellular processes and pathways and plays a critical role in snRNP complex assembly in the cytoplasm. Ninety-five percent of SMA patients have a homozygous deletion of exon 7 of *SMN1* or gene conversion from *SMN1* to *SMN2* (5q-SMA patients) [10], while the remaining 5% are compound heterozygotes for a deletion of exon 7 in SMN1 and a point mutation. In order to obtain a new view and future perspectives of SMA, in this review we discuss different aspects of this severe neuromuscular disorder: the historical perspective, from the early description and manifestations to the molecular genetic characterization, the clinical features and its classification into five types, the diagnostic process, the psychological outcome, recent innovation in treatments and therapies, the possibility of an early identification of affected infants in the pre-symptomatic phase through newborn screening programs.

## 2. History of Spinal Muscular Atrophy

**Figure 1:**
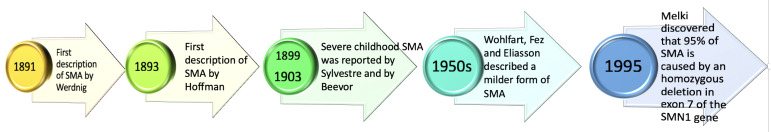
SMA timeline. SMA was first reported by Werdnig in 1891 and then by Hoffmann in 1893 [10]. In 1899 and 1903, Sylvestre and Beevor recognized variability of muscle weakness severity [11]. In the 1950s, Wolhfart, Fez, and Eliasson described a milder form of SMA [11] and in 1995 Melki discovered that 95% of SMA cases are caused by a homozygous deletion in *SMN1* [13].

SMA, an inherited, progressive neuromuscular disease that can cause muscle atrophy, was first discovered in infants in the early 1890s by physicians Guido Werdnig and Johann Hoffman. Werdnig meticulously described the disease in two newborn siblings in 1891 and Hoffmann characterized what he used to term “infantile progressive muscular atrophy” and detailed the clinical presentation in seven patients from 1893 to 1900 [11]. In their papers, Werding and Hoffman were able to provide a rather complete picture of the clinical and pathologic aspects of infantile SMA [11]. Although the etymon of Werdnig–Hoffmann disease was later attributed to the severe childhood form of SMA, their cases were of moderate severity. The first descriptions of severe childhood SMA were reported by Sylvestre in 1899 and by Beevor in 1903 [12]. It was only in the 1950s that Wohlfart, Fez, and Eliasson described a milder form of SMA in which patients could maintain the ability to stay upright and to ambulate [12]. All cases described throughout the years showed an anterior horn cell degeneration and symmetrical, proximal predominant extremity weakness that also affects axial, intercostal, and bulbar musculature [12]. At the International Consortium on Spinal Muscular Atrophy, the different phenotypes described were classified into three types of SMA based on motor function and age of disease onset [13]. Later, a fourth category was added for adult-onset cases and a zero category for patients with pre-natal onset and death within the first few weeks of life. However, 25% of patients still elude a well-defined classification of the disease. The classification of SMA presented a mystery regarding the severity of the disease, namely how a genetic defect could cause a wide range of more or less severe phenotypes. That enigma was finally solved in 1995 in Melki’s laboratory, where it was discovered that 95% of SMA cases are caused by a homozygous deletion in exon 7 of the *SMN1* gene, on chromosome 5q13.3 [14]. Figure 1 represents in a timeline the highlights in the history of SMA from the first reports and descriptions to the classification of different forms.

In addition, in humans, there are two forms of the *SMN* gene: the telomeric form (*SMN1*) and the centromeric form (*SMN2*). The *SMN1* gene produces the encoding SMN protein and the *SMN2* gene is identical to *SMN1* except for a nucleotic substitution at position 840 of a cytosine to a thymine that excludes exon 7 from transcription. In particular, the exclusion of exon 7 from the transcription process is not complete, and only a small portion of the total mRNA transcripts (10–15%) of the *SMN2* gene contain exon 7 encoding a normal SMN protein. SMA patients have a non-functional *SMN1* gene, and therefore the *SMN2* gene plays a crucial role in the production of SMN protein. Therefore, SMA is a condition caused by a deficit in the SMN protein which causes selective motor neuron loss. Disease severity is explained by *SMN2* copy variability but it is not the only phenotypic modifier [15,16]. Studies conducted in animal models have significantly contributed to the development of therapies for SMA. For example, Monani et al. conducted studies in mouse models. Mice lacked the *SMN2* gene and consequently the deletion of the *SMN* gene was lethal. Monani et al. found that insertion of two copies of human *SMN2* into mice caused the development of a severe SMA-like phenotype with motor neuron loss. In contrast, mice with eight copies of *SMN2* were normal [17]. These models played a crucial role as they allowed molecular and biochemical studies and initiation of the development of therapies aimed at increasing SMN protein expression and preventing motor neuron loss [17].

## 3. Clinical Features of SMA

SMA is a monogenic, autosomal recessive neuromuscular disorder, characterized by muscle weakness and atrophy caused by degeneration of alpha motor neurons of the anterior horns of the spinal cord.

The weakness usually affects the lower limbs with diffuse areflexia. The onset of weakness ranges from birth to adulthood and is symmetric, proximal to distal, and progressive. Facial and ocular muscles are usually unaffected. SMA has been classified into five types based on severity of symptoms, age at onset, highest motor milestone achieved, and genotype (Table 1) [18]. Usually, the severity of clinical manifestations is inversely proportional to *SMN2* copy number. *SMN2* can only partially compensate for the loss of *SMN1*, resulting in a deficiency but not a complete depletion of SMN protein [19]. Literature data have shown that patients with SMA have normal or above-average cognitive abilities [20,21].

SMA type 0 (SMA 0) is congenital, and presents at birth as severe weakness, hypotonia, and respiratory distress. There may be a history of restricted movements in utero, joint contractures, areflexia, and atrial septal defect. Patients with SMA 0 have severe respiratory diseases and die within the first six months of life [16]. SMA 0 patients have a single copy of *SMN2*.

SMA type 1 (SMA 1) is the most common form of SMA (50–60% of cases) and manifests early in the first six months of life, with severe weakness and generalized hypotonia, poor motor abilities, areflexia, and feeding, swallowing, and breathing difficulties with premature death within 24 months [24]. The mean age at onset of symptoms is 2.5 months. Affected children may acquire head control and ability to roll, but then lose these abilities before the age of six months. They have a characteristic “bell-shaped” chest and abdominal breathing due to the weakness of the intercostal respiratory muscles with preservation of diaphragmatic muscles. There may also be fasciculation of the tongue [16]. SMA 1 patients typically have one to two copies of *SMN2* [25].

SMA type 2 (SMA 2) patients usually manifest motor symptoms after the first 6 months of life. Children with SMA 2 may reach motor milestones slowly and, with supportive care only, they can achieve the ability to sit independently. Moreover, they may stand with support, but they are never able to walk without any help [22]. Common clinical manifestations include progressive proximal muscle weakness, lack of muscular tone, hand tremor, reduced or absent deep tendon reflexes, scoliosis, and progressive respiratory muscle weakness that results in a restrictive lung disease, potentially leading to death. These patients represent approximately 30% of cases and they usually have three copies of *SMN2* [2].

SMA type 3 (SMA 3) symptoms begin after 18 months of age and are heterogeneous. Patients present a progressive proximal muscle weakness, and the legs are more severely affected than the arms [25]. SMA 3 can be subdivided into type 3a (clinical symptoms before the age of three) and type 3b (clinical symptoms after the age of three). The children generally reach the major milestones, including independent walking, but their motor performance levels vary widely. Some children are barely able to get up from a sitting position and walk a few steps without help, while others walk well and can climb stairs [27]. SMA 3 accounts for about 10% of the patient population and most of these have three or four copies of *SMN2* [2].

SMA type 4 (SMA 4) is the least common form of SMA (<5% of cases), and the life expectancy is normal [16]. SMA 4 was added to this classification to describe patients with onset in adulthood (>18 years) and mild course. This group includes patients who can walk in adulthood and have no problems with breathing and feeding [26]. *SMN2* copies are usually between three and five [25].

## 4. SMA Diagnosis with Molecular Genetic Testing

Since 1995, when Lefebvre identified the SMA-determining gene [14], several diagnostic genetic tests have become commercially available to identify individuals with SMA. Many DNA tests are available to detect the absence of exon 7 of the *SMN1* gene, used as a diagnostic test for SMA patients. According to a recent study [28], determining the exact copy number of the *SMN2* gene is crucial to assess eligibility for treatment, so advanced techniques are needed to discriminate *SMN1* from *SMN2* [29]. Many techniques are used to detect copy number variations, but most cannot detect single-exon deletions or duplications. Current molecular methodologies for the detection and diagnosis of SMA by revealing *SMN1* deletion and by *SMN1* and *SMN2* gene quantitation, respectively, include single-strand conformation polymorphism, restriction fragment length polymorphism, real-time polymerase chain reaction (RT-PCR), denaturing high-performance liquid chromatography, multiplex ligation probe amplification (MLPA), quantitative PCR (qPCR) and competitive PCR, high-resolution melting analysis, and liquid microbead assay [8]. More recently, digital PCR has shown a growing range of applications, being used not only to identify SMA individuals but also to determine *SMN1* and *SMN2* copy number [8]. As Mercuri et al. reported, the gold standard of SMA genetic testing is a quantitative assay of *SMN1* and *SMN2* using MLPA or quantitative polymerase chain reaction (qPCR) [30]. The qPCR is an assay that accurately quantifies *SMN1* and *SMN2* exon 7 copy number and *SMN1* gene duplication. There are commercial kits that are engineered to detect certain gene conversions from *SMN1* to *SMN2* and *SMN2* to *SMN1* by checking the sequence identity of exon 7 and intron 7. These kits also can determine the presence of certain alleles associated with gene duplication [29] and enhanced splicing of *SMN2* [31]. The disadvantages of multiplex qPCR include: (1) the use of genomic DNA (gDNA) isolated from whole blood collected in EDTA tubes or buccal swab with a concentration of 10–40 ng/µL, processed within 14 days after isolation; (2) the inability of detecting nonsense, frameshift, or missense mutations; (3) the distinction between samples with two copies of *SMN1* on one chromosome and zero copies on the other (2 + 0 or healthy carriers) and samples with one genomic copy of *SMN1* on each chromosome (1 + 1) can be carried out only by analyzing the genotype of gene duplication variants in certain populations [29]; (4) determining the gene copy number for all *SMN* exons, useful for rare cases of SMA with complicated gene structures, is not possible; (5) furthermore, the primers used in this technique have binding sites not containing polymorphic sites with minor allele frequencies (MAFs) greater than 0.005 (according to the Single Nucleotide Polymorphism Database (dbSNP) build 152). However, very rare polymorphisms located within the primer-binding sites may potentially impact the accurate quantification of the number of *SMN1* and *SMN2* copies [23]. Despite these disadvantages, the multiplex qPCR assay has the lowest cost and shortest duration (<4 h). Thus, results have demonstrated that the multiplex qPCR assay is rapid, accurate, and cost-effective and represents a high-throughput strategy. Considering the low cost, the high-throughput approach, and the high accuracy of the multiplex qPCR assay, it can be considered a routine tool for clinical diagnosis and for screening of SMA carriers. MLPA, on the other hand, is a multiplex PCR-based method using a single primer pair to amplify and quantify specific and multiple genomic loci (20 independent control loci for *SMN1/2*), in a single tube reaction from only 20 ng of a patient’s genomic DNA. MLPA technology allows the simultaneous detection of *SMN1* and *SMN2* copy numbers, thus facilitating diagnosis. The MLPA technique is more sensitive and allows a high degree of precision for the quantitative detection of three *SMN1* copies or fewer. Commercial kits of probe sets are available and provide different SMA assays, fitting the genetic testing needs [32,33].

MLPA technology has several important limitations, including: (1) DNA sequence variants located in probe-binding sites of *SMN1* alleles may interfere with probe hybridization and result in a false-positive carrier (one copy) or false-positive diagnostic (zero copies) result; (2) reactions are sensitive to contaminants but generate uninterpretable results; (3) MLPA cannot yet be used to investigate single cells, important for pre-implantation genetic diagnosis testing; (4) MLPA is not a suitable method to detect unknown point mutations; (5) MLPA probes are sensitive to small deletions, insertions, and mismatches; (6) MLPA requires a CE analyzer, which is a higher-cost option compared with slab gel electrophoresis for RFLP [23]. Advantages and disadvantages of multiplex qPCR and MLPA as molecular genetic tests for SMA diagnosis are summarized in Table 2.

## 5. Psychological Adjustment of Individuals with SMA

The psychological adjustment of individuals with SMA is challenging to discern due to limited quantitative research employing standardized instruments that focus on the subjective well-being of individuals with SMA. This situation arises from the difficulties inherent in conducting research within this specific context [34]. Nevertheless, previous research has explored various aspects of SMA individuals’ lives, including their quality of life, acceptance of the disease, self-esteem, emotions, and social roles across different age groups including adults, adolescents, and children. These studies investigated mostly the experience of individuals with SMA 3 and SMA 4, due to the difficulty of individuals with SMA 1 and SMA 2 in reaching adult age. In the last two cases, research analyzed the families’ experiences and psychological burden [35].

Regarding adult and adolescents, Wan et al. [36] conducted a qualitative research study on psychological well-being of individuals with SMA and the impact of the disease, identifying four recurring themes in their experiences: distress in response to changes in physical function, the influence of stigma on participants’ social expectations, resilience, and grit in the face of challenges posed by the illness. Another study showed that, despite the physical limitations imposed by their disease, participants generally reported satisfaction with their overall life [35]. Furthermore, it was noted that sociodemographic characteristics and clinical variables, such as motor function, were unrelated to psychological well-being, except in the case of females, who exhibited a higher susceptibility to negative emotions [34]. In terms of self-esteem, participants in this study displayed high self-esteem scores [34].

A recent study among adults highlighted that individuals with SMA perceived their health-related quality of life to be very similar to reference values of the general population except for the domain of physical functioning [37]. Conversely, a comprehensive American study suggested that perceived quality of life of respondents with SMA was lower in all domains compared to healthy subjects [38]. As a result, studies investigating the impact of illness severity on quality of life in individuals with SMA have reported contradictory findings. Other variables appear to play a significant role in psychological adjustment of individuals with SMA. It has been observed that disease acceptance is a correlate or predictor of subjective well-being among individuals with chronic physical disabilities [39,40]. A study among adults with various physical disabilities underscored that acceptance of the illness was positively associated with self-acceptance, well-being, positive relations, family satisfaction, and the presence of meaning, defined as the extent to which individuals grasp, make sense of, and find significance in their lives [41,42]. Indeed, Zhang et al. [43] noticed that the acceptance of one’s disease denotes the degree to which individuals integrate their lifestyle into the experience of dealing with the disability.

Regarding social relations, a study involving young individuals highlighted that social support from family, but not from friends, was significantly associated with better psychological adjustment [44]. The authors found significant associations between family support and age, as well as between friend support and motor functioning, in predicting functional ability [44]. Another study emphasized the importance of social relationships [36]. Wan and colleagues [36] reported that individuals with SMA often received informal support from family, friends, and peers to fill gaps in formal care. However, practical support provided by family and friends was seen as unsustainable over time. In general, social support from family and friends was reported as a facilitator for coping with the challenges of illness [45,46]. Furthermore, Fischer et al. [34] reported that adults with SMA were highly satisfied with their participation in social activities. Their research showed that societal participation explained 30–50% of the variance in psychological well-being [34]. Considering all these aspects, it can be argued that the acceptance of disability and social relationships can improve autonomy, perceived competence, and overall quality of life. Additionally, it could be hypothesized that these factors create a positive cycle, wherein individuals with a higher sense of well-being are more motivated to be active and to engage in social activities, and vice versa [34]. As Post et al. [47] suggested, well-being and mental health are closely related to an individual’s level of participation. Conversely, individuals with SMA typically rely on assistance from others, such as caregivers, to engage in various activities. This condition also prompts reflection on the caregivers who take care of individuals with SMA. A recent review on this topic highlighted that most studies have reported decreased quality of life, as well as moderate to high levels of burden and distress among caregivers of individuals with SMA. Another review conducted on parents of children with SMA 1 and 2 showed that parents feel sad, helpless, hopeless, and frustrated about their child’s future, due to the fact that they might lose their children at any time [35].

To alleviate the burden on families and facilitate the participation of individuals with SMA in social activities, healthcare policies should address the needs of families and provide support for caregiving, decision making, and activity organization [48], as has also been shown for other categories of chronic disease patients [49].

Regarding emotional states, some studies found an association between less severe physical limitations, such as ambulation, physical decline following long periods of stability, and increased emotional distress [37,50,51]. Subjective feelings of depression and anxiety were generally low in adults with SMA [52]. However, for school-aged individuals with SMA, there was a high prevalence of anxiety and depression [53]. In a research study involving children and adolescents aged 8–18, high levels of anxiety and depression were reported, and these were associated with factors such as respiratory system dysfunction, digestive system dysfunction, skeletal deformity, rehabilitation exercises, academic delay, specialized support from schools, household income levels, caregivers’ subjective anxiety, and caregivers’ expectations [53].

## 6. SMA Treatments and Therapies

As SMA is a systemic disease, interdisciplinary management of respiratory, nutritional, gastroenterological, orthopedic, and psychosocial problems is required to care for patients with SMA. In 2007, general treatment recommendations were addressed and published in the first consensus statement pointing out the standards of care for SMA. However, the implementation of these standards of care can vary significantly depending on different factors such as cultural perspectives, socioeconomic aspects, and the availability of resources [54,55].

In the past decade, the introduction of new therapies has led to a significant change in the SMA treatment landscape. Today, multiple types of treatments are available for SMA, including *SMN2* splicing modifiers and gene replacement therapy. Clinical studies have demonstrated the potential of both these therapy approaches to positively alter the course of SMA in humans [30,56].

### 6.1. FDA-Approved SMN-Based Therapies for SMA

In the absence of therapeutic intervention, the *SMN2* gene produces mostly truncated SMN protein and only a small fraction of full-length SMN protein, due to poor inclusion of exon 7 in mature mRNA transcripts. However, FDA-approved therapies are aimed at increasing the inclusion of exon 7 in mature mRNA transcripts to enhance the production of full-length SMN protein [57].

FDA-approved therapies for SMA, including nusinersen, onasemnogene abeparvovec, and risdiplam, have improved motor function and lifespan for many patients. However, not all patients respond optimally, particularly those with one copy of *SMN2* or those who receive post-symptomatic treatment. Furthermore, cost, availability, access, and the patient’s condition may limit the effectiveness of SMN-based therapy. Thus, SMN-independent strategies to improve motor function and quality of life are needed [58,59].

**Figure 2:**
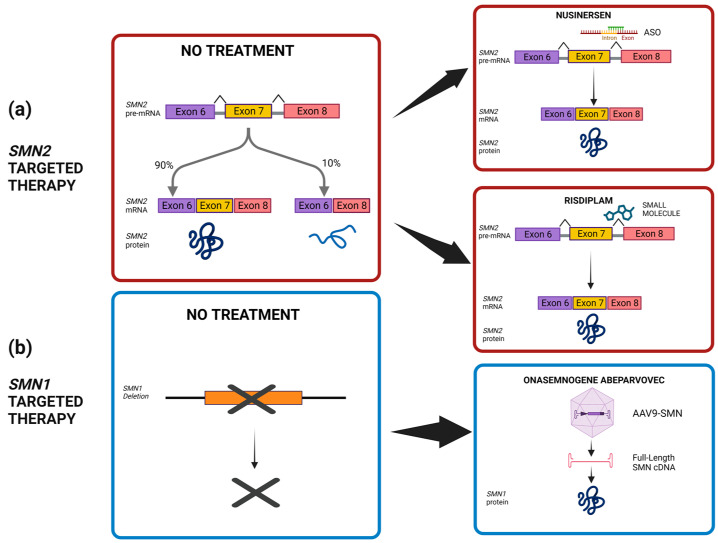
FDA-approved SMN-based therapies for SMA and their mechanism of action [19,67]. (**a**) The different actions of *SMN2* splicing modifiers such as nusinersen (ASO) and risdiplam (small molecules in comparison to the absence of therapeutic intervention). Both *SMN2*-targeted therapies promote the inclusion of exon 7 in mature mRNA transcripts. (**b**) The mechanism of *SMN1*-targeted therapy, by non-replicating self-complementing AAV-9, compared to the homozygous loss of functional *SMN1* gene. The onasemnogene abeparvovec gene therapy carries full-length human *SMN* cDNA to guarantee the production of SMN functional protein.

The first FDA-approved therapy for SMA was nusinersen (trade name Spinraza). The arrival of nusinersen in 2016 marked the first drug capable of altering the natural history of SMA in the United States, which followed in Europe the year after: the drug was approved in 2016 by the FDA and in 2017 by the EMA for all subtypes of 5q-SMA patients [60,61]. Nusinersen is an oligonucleotide based on antisense technology and an *SMN2* splicing modifier able to alter the splicing process of *SMN2* messenger RNA. This alteration leads to the production of a functional protein in the central nervous system (Figure 2a). However, it must be administered intrathecally, meaning it is directly injected into the fluid surrounding the central nervous system [60,61]. This invasive practice can only be performed in a hospital setting. Despite the inconvenience of the method of administration, nusinersen has been shown to increase survival without permanent respiratory support in SMA 1 and has increased motor function development in types 1–3 [62]. However, improvements in SMA 2 and 3 were less evident [63,64]. Studies have shown that, if administered before the onset of symptoms, nusinersen may result in near-normal motor development in children with SMA [65,66].

Nusinersen has been associated with several adverse events, with the majority being pyrexia, upper respiratory tract infections, nasopharyngitis, vomiting, headache, and constipation. Serious adverse events were also reported, which were in line with the typical nature and frequency of events observed in the context of SMA or during lumbar puncture procedures [22,68]. In the past, a variety of side effects have been identified for different antisense oligonucleotides (ASOs). Thus, it is important to mention that, compared to other ASOs, nusinersen is safe and there is no evidence of clinically relevant problems [69]. However, nephrotoxicities as side effects on the kidney, including renal tubular degeneration, glomerulonephritis, and increased urinary protein levels, are a potential risk in nusinersen-treated patients [70]. Screening for SMA may lead to early detection and facilitate prompt treatment using nusinersen [71,72].

In March 2021, Italy received a new gene therapy drug called onasemnogene abeparvovec (Zolgensma). This drug was approved by the FDA in July 2019 and by the EMA in May 2020. It is intended for patients with symptomatic 5q-SMA type 1 SMA under six months of age and for pre-symptomatic 5q-SMA patients with 2–3 copies of *SMN2*. While it is reimbursable up to 21 kg in Europe, in Italy, it is only compensated up to 13.5 kg and up to two copies of *SMN2*. However, individuals with three copies of *SMN2* who are symptomatic are still eligible [73,74].

Onasemnogene abeparvovec is a non-replicating self-complementing adeno-associated serotype 9 (AAV-9) viral vector that carries full-length human *SMN* cDNA controlled by the hybrid CMV enhancer/chicken β-actin promoter. The single intravenous administration over a 60 min period allows the vector to cross the blood–brain barrier into the central nervous system where it is endocytosed by cells, including motor neurons, and transduces host cells to transcribe its double-stranded DNA unit [57] (Figure 2b). Prior to and after infusion of onasemnogene abeparvovec, it is recommended to initiate an immunomodulatory regimen with corticosteroid administration to reduce the risk of side effects [75]. Serotype AAV9 targets cells efficiently and important improvements have been shown in motor function for SMA-affected individuals treated pre-symptomatically. Of interest, research studies in porcine and macaque models highlighted that post-symptomatic administration may have limited benefit because of inability to recover motor neurons [19,74]. Moreover, clinical trials demonstrated that affected individuals with three copies of *SMN2* were able to reach motor abilities according to age and that the loss of previously gained motor skills are prevented by post-symptomatic treatment. However, less than half of SMA-affected patients treated post-symptomatically reach advanced motor skills such as walking or standing [76,77]. Furthermore, it has been shown that onasemnogene abeparvovec significantly improved airway function, crucial for SMA-affected individuals requiring permanent ventilation therapy [78]. However, the risk of neutralizing antibodies of maternal origin, serious liver complications, as well as the cost of the single-dose gene therapy are all potential drawbacks of this treatment [19].

Before infusing onasemnogen abeparvovec, it is recommended to test patients for the presence of anti-AAV9 antibodies. The half-life of IgG antibodies acquired passively is about 35 to 40 days. In general, in children, the level of antibodies may reflect maternal placental transfer, especially in infancy [79]. Therefore, a follow-up test for antibodies is strongly suggested, mainly in the case of positivity in the initial assay, within f two weeks to enable the decrease in moderately elevated titers to an acceptable level before treatment. It is important to specify that infants with an antibody titer above 1.5 cannot receive treatment, although only approximately 6% of infants present this titer [75,80]. As already described in clinical trials, the most common adverse reactions associated with onasemnogene abeparvovec were elevated aminotransferases and vomiting [81]. Moreover, warnings as indicated in the prescribing information are acute serious liver injury, elevated aminotransferases, elevated troponin-I, and thrombocytopenia. Following the rare occurrence of acute liver injury, serial monitoring of liver function, platelets, and troponin-I concentrations is recommended [81]. Following onasemnogene abeparvovec therapy, it is also important to mention the rare occurrence of severe adverse events, like thrombotic microangiopathy and atypical hemolytic uremia syndrome [81,82].

On 1 April, the European Commission gave the green light to a new drug, risdiplam (commercial name Evrysdi), which can modify diseases. It is the second splicing modifier approved by the FDA in July 2020 and designed for patients with SMA types 1, 2, or 3 with 5q-SMA and of two months of age and older. Being the only orally administered drug, families showed a lot of interest [83]. Unlike Spinraza, risdiplam is effective on the entire body, not only on the central nervous system [84] (Figure 2a). Research studies comparing Evrysdi with Spinraza showed that Evrysdi is indeed a valid alternative for SMA 1, as it can improve survival rates and motor function. However, it is important to note that, while Evrysdi can be used to treat patients as young as two months old, it is not necessarily the preferred choice of clinicians for patients diagnosed by a neonatal screening program and possibly treated before the age of two months [85,86]. Details of each disease-modifying therapy (DMT), as described above, are summarized in Table 3. Moreover, Figure 2 illustrates the mechanism of action of the FDA-approved SMN-based therapies for SMA.

### 6.2. Neuroprotective Drugs

Neuroprotective strategies aimed at preventing dysfunction in motor neurons and associated circuits, but independently of survival motor neuron (SMN), have been attempted for SMA with limited success [19]. Although neuroprotective treatments have been used in other neurodegenerative diseases, such as Alzheimer’s and Parkinson’s disease, the complexity of the process of neurodegeneration suggests that targeting single-cell pathways may not be enough to halt or improve disease progression. Therefore, neuroprotective strategies would likely require SMN-targeted treatments to be effective. Combining neuroprotective strategies with SMN-dependent approaches could maximize therapeutic benefits [87].

### 6.3. Neuromuscular Junction Drugs

Targeting calcium homeostasis in developing motor nerve terminals can improve neuromuscular dysfunction and motor capacity. Neuromuscular activity drives muscle strength, endurance, and motor skills, which are crucial for daily activities such as wheelchair mobility, food preparation, hygiene, and computer use. Complementary treatments targeting neuromuscular junction function can improve motor skills and quality of life [19].

In conclusion, improving motor skills is essential for SMA patients to perform daily activities independently, enhancing their quality of life and reducing caregiver burden. It is worth emphasizing that the combination of neuroprotective strategies with SMN-dependent approaches and complementary treatments targeting neuromuscular junction function could maximize therapeutic benefits for SMA patients [19].

Most importantly, multidisciplinary management involving physiatrists, orthopedists, and physiotherapists is necessary to prescribe the aids to maintain the patient’s motor autonomy. Periodic and continuous monitoring by a nutritionist, pulmonologist, and neurologist are also important.

## 7. Newborn Screening for SMA

### 7.1. New Perspectives on SMA through Newborn Screening

SMA is a severe and progressive neuromuscular disease, the most common genetic cause of infant mortality, characterized by loss of motor neurons causing muscle weakness and atrophy [1,2,3,4,5]. Historically, diagnostic delay has always been a challenge for this rare and devastating disease, but, following improvement in supportive therapies and the availability of DMTs, a new scenario for SMA has been emerging, leading to a breakthrough in the natural history of affected children [88,89]. In fact, as detailed above, three DMTs have been approved by the U.S. Food and Drug Administration (FDA) since 2016: nusinersen in December 2016, onasemnogene abeparvovec in May 2019, and risdiplam in August 2020 [88]. Both pre-clinical trials in mouse models and clinical data demonstrated the importance of early and pre-symptomatic treatment in modulating the rapid and progressive degeneration associated with SMA [88,90,91,92]. Considering that the best outcomes have been described when treatments start as early as possible, hopefully before significant motor weakness or loss occur, time of diagnosis and treatment is crucial for SMA [8,90]. Of note, it has already been reported that motor neuron loss in SMA 1 patients is an irreversible process and begins in the peri-natal period, leading to a severe denervation in the first three months and loss of over 90% of motor units at 6 months [91]. With these recent advances in treatment aiming to increase the expression levels of SMN protein, the possibility to identify SMA-affected infants early in the pre-symptomatic phase through newborn screening (NBS) programs represents an imminent need and is essential for the achievement of maximal therapeutic benefit [2]. It is also important to consider that NBS for SMA gives the unprecedented opportunity to obtain the maximal benefit from these therapies without increasing the cost of treatment since most affected patients still need treatment once diagnosed [2]. As different authors already discussed, SMA can be recognized as “one of the golden candidates” for inclusion in NBS panels [89,93,94]. The idea to include SMA in NBS programs is strongly supported by the fulfilment of the Wilson and Jungner criteria and by experts that agree to establish NBS for SMA [95,96]. The ten principles for population-based screening decisions, outlined in a seminal WHO publication entitled “Principles and Practice of Screening for Disease” in 1968 [97], include considerations on the disease itself (prevalence, severity, natural history), the availability of an effective treatment and of a reliable screening test, and societal considerations (cost/effectiveness, false-positive results) [93]. Therefore, in recent years, thanks to national or regional pilot projects, several NBS programs have been implemented including a screening test for SMA in many countries. As detailed by Dangouloff et al. [98], SMA NBS was implemented in different countries. Australia [99,100], Belgium [101], Canada [93,94], Germany [102,103], Italy [89], Japan [104,105], Taiwan [106], and the United States [107] screen for SMA, in part of the country or in the whole country. In fact, the Advisory Committee on Heritable Disorders in Newborns and Children (ACHDNC) added NBS for SMA to the Recommended Uniform Screening Panel (RUSP) in July 2018 [98,103]. Of interest, as specified by Abiusi et al., the first Italian SMA NBS project showed the highest incidence reported so far (1:6059) [89].

### 7.2. Molecular Analysis as Newborn Blood Screening Test for SMA

As already discussed above, NBS for SMA started in many countries and regions as a pilot project. For this reason, ethical committee approval was required before implementing SMA NBS [98]. However, no biochemical marker has been identified to be used as a clinically meaningful biomarker in NBS [8,108] and the NBS assay is based on a very sensitive, accurate molecular testing with a high predictive power. The assay consists of a real-time PCR analysis able to detect the presence of the *SMN1* gene from a dried blood spot (DBS) sample, more precisely by identifying homozygous deletions of exon 7 in the *SMN1* gene in affected infants. As for routine pre-existing metabolic NBS workflow, DBS samples for SMA NBS are collected by pricking the newborn’s heel within 48–72 h of birth and by letting the drops of whole blood dry on a special filter paper card, better known as a Guthrie card. Having started as a pilot project and not being mandatory by law in many regions or countries, details on the SMA NBS pilot via informative sheets and consent forms are provided to all parents before sample collection. More precisely, local health personnel provide all the necessary information about SMA NBS to families. Then, neonatal DBS samples are sent to the NBS reference laboratory within 24–48 h from their collection. Most NBS laboratories perform testing on the day of DBS sample receipt or within 48 h of their arrival. The molecular screening test is usually performed on a 3.2 mm disk (approximately 3–3.2 μL whole blood) punched out from the DBS specimen, followed by DNA extraction.

The protocol designed for NBS is very quick and provides results in a very short time. It consists of two main steps consisting of DNA extraction and amplification analysis by real-time PCR. In particular, the SMA primary screening test allows the qualitative detection of exon 7 in the *SMN1* gene.

**Figure 3:**
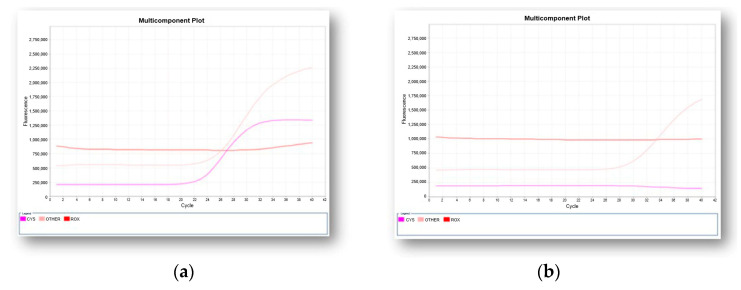
Two representative real-time PCR multicomponent plots obtained by the qualitative detection of exon 7 in *SMN1* gene. Internal control (pink curve), *SMN1* gene (purple curve): (**a**) a negative result with the amplification of the *SMN1* gene is shown; (**b**) a positive result as determined by the absence of amplification of *SMN1* gene is reported.

A valid negative result for SMA is determined by amplification of the *SMN1* gene (Figure 3a) while a valid positive result is determined by the absence of amplification of the *SMN1* gene (Figure 3b).

**Figure 4:**
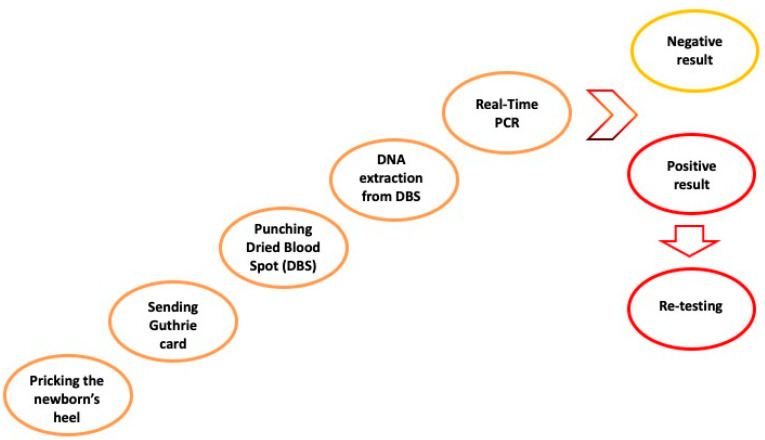
SMA NBS workflow [6,53,57].

In case of a negative SMA NBS result, no further action is needed, and no information is provided to families, as already agreed in NBS policy [89]. To confirm the positive result, the sample analysis must be briefly repeated. For a confirmed positive SMA NBS test, it is necessary to proceed with the recall of the newborn and confirmatory testing analysis. Most importantly, to provide information regarding SMA, on the significance of the positive test result, and to conduct a careful clinical evaluation of the infant, multidisciplinary counselling with medical geneticists, pediatric neurologists, and also a psychologist is essential for the family [89]. Figure 4 represents the SMA NBS workflow.

The molecular test for SMA NBS is inexpensive and, as has already occurred for other NBS analyses, it can be multiplex [2,109]. Bearing in mind the paramount need for a screening test for SMA, commercial real-time PCR assay kits have been developed by different companies. These include: Eonis™ SCID-SMA kit (PerkinElmer, Turku, Finland), SPOT-it™ TREC & *SMN1* Screening Kit (ImmunoIVD, Nacka Strand, Sweden), NeoNat SCID-SMA REAL-TIME PCR KIT (Labsystems Diagnostics Oy, Vantaa, Finland), Targeted qPCR SMA and Targeted qPCR SMA FLEX (Zentech, Liège, Belgium), SALSA MC002 SMA Newborn Screen (MRC Holland, Amsterdam, the Netherlands), TaqMan™ SCID/SMA Assay (Applied Biosystems™, Thermo Fisher Scientific, Waltham, MA, USA). Some of the SMA assay kits are certified for use in diagnostic procedures (in vitro diagnostic, IVD), while some are for research use only (RUO). Anyway, most of the commercial kits available for SMA are all multiplex and intended for simultaneous screening of SMA and severe combined immunodeficiency (SCID) in newborns.

## 8. Discussion

In recent years, advances in treatment and care for SMA-affected patients significantly changed. As well known, SMA has historically been recognized as the leading cause of hereditary death in infancy, worldwide. However, thanks to three newly FDA-approved DMTs, implementation of NBS programs, and consequently the possibility of an early intervention, there is a reasonable opportunity to greatly and rapidly change the natural history of the disease [2,10]. In recent years, SMA NBS pilot projects not only pointed out a higher incidence of the disease than expected, but also demonstrated that the possibility to identify infants with SMA in the neonatal period leads to improved outcomes when SMA-specific DMTs are promptly provided. As Vill et al. assessed, pre-symptomatic therapy prevents the death of motor neurons, considering that in their study all pre-symptomatically treated affected individuals, even with two *SMN2* copies, achieved normal motor development [103]. It should be recognized that determining the copy number of *SMN2* in the diagnostic confirmation process is certainly a key step in defining the most suitable therapeutic option. At the same time, it is also a very delicate phase. Moreover, recommendations for the treatment of SMA patients soon after their identification by an NBS program are complicated by different barriers including administrative, institutional, and insurance-related ones. This aspect is very important when remembering that SMA infants identified by NBS tests should be potentially treated within 14 days of life [10]. In this review, the basic criteria for the inclusion of a rare disease in NBS programs have been reported, and the benefits of SMA NBS have also been discussed. As is well known, cost factors for SMA NBS represent a critical issue but, at the same time, a decisive factor [103], and only very preliminary health economic data or cost-effectiveness data for SMA NBS are available [98]. In this context, it is worth mentioning the Italian situation: in the last five years, many regions in Italy implemented NBS for SMA, particularly thanks to effective DMTs available and reimbursed by the National Health Service (SSN). For this reason, Ghetti G et al. started an evaluation study of the cost-effectiveness of SMA NBS in Italy, demonstrating that NBS, when followed by pre-symptomatic treatment for SMA, results in good value for money and is cost-effective for the Italian SSN [110].

Importantly, as rightly argued by Abiusi et al., the spread of SMA NBS will point out the need to define accurate and univocal guidelines as well as standard analytical procedures for SMA molecular testing, both for primary screening tests and diagnostic confirmation analysis [89]. It is noteworthy that in the coming years the identification of SMA patients will be fulfilled by molecular testing rather than by a clinical picture, leading to the impellent demand for rewriting the natural history of this severe genetic neuromuscular disease as well as the standards of care [89].

Following this line, even the psychological support in the NBS context will change, and it should be addressed to parents. In most cases, parents tend to be dissatisfied with the quality and depth of the information received and prefer in-person visits, finding them clearer, more welcoming, and reassuring [111]. In particular, they need a caring, empathic, and safe setting of communication [111]. It will also be important for health professionals to improve communication resources to mitigate the impact of positive screening results and to offer psychosocial interventions to support families in the future [112].

In conclusion, the new scenario for SMA, following implementation of NBS programs and the availability of recently approved DMTs, which together ensure an early intervention, reveals an urgent need: the identification of clinically meaningful biomarkers, as a follow-up tool, essential to measure and evaluate the disease across time. Therefore, many efforts are being made to define SMA biomarkers of importance to highlight the underlying mechanisms of the disease but also to detect the disease progression, allowing for more appropriate and personalized timing and dosing of therapy for affected patients [108].

## Figures and Tables

**Table 1:** Classification of SMA into five types based on symptom severity, age at onset, highest motor milestone achieved, and genotype [2,19,21,22,23,24,25,26].

SMA Type	Age of Onset	Motor Milestones	Other Characteristics	Life Expectancy	*SMN2* Copy Number
SMA 0	Pre-natal	None reached	Reduced movement in utero Severe neonatal weakness and hypotonia Areflexia Respiratory failure at birth Joint contractures Facial diplegia Atrial septal defects	<6 months	1
SMA 1	0–6 months	Some head control, sits with support	Poor head control Paradoxical breathing Muscle weakness and hypotonia Areflexia or hyporeflexia Variable suck and swallow difficulties	<2 years	2
SMA 2	6–18 months	Sits, never stands independently	Developmental delay with loss of motor skills Hyporeflexia Proximal muscle weakness Postural tremor or fingers	>2 years	2–3
SMA 3	>18 months	Walks	Proximal muscle weakness Loss of motor skills Fatigue Postural tremor or fingers Loss of patellar reflexes	Adult	3–4
SMA 4	Adulthood	All motor functions	Very mild but progressive muscle weakness Fatigue	Adult	≥4

**Table 2:** Advantages and limitations of multiplex qPCR and MLPA as molecular genetic tests for SMA diagnosis [22,23,24,25].

Technique	Advantages	Limitations
**multiplex qPCR**	(1) Detection of the presence of c.859G > C polymorphism in *SMN2* gene (2) Low cost (3) Short work time (<4 h) (4) High throughput (5) High accuracy	(1) DNA sample from whole blood collected in EDTA tubes or buccal swab (ranges from 10–40 ng/microL) (2) No detection of nonsense, frameshift, or missense mutations (3) Discovery of silent carriers only from the genotype of gene duplication variants in certain populations (4) No determination of copy numbers for all *SMN* exons (5) Polymorphisms located within the primer-binding sites may potentially impact the accurate quantification of the number of *SMN1* and *SMN2* copies
**MLPA**	(1) Detection of copy number for all *SMN* exons (2) DNA sample from whole blood, pre-natal samples, and dried blood spot cards (3) High precision for the quantitative detection of three *SMN1* copies or fewer	(1) DNA sequence variants located in probe-binding sites of *SMN1* alleles may interfere with probe hybridization (2) Reactions are sensitive to contaminants (3) Cannot yet be used to investigate single cells, which is important for pre-implantation genetic diagnosis testing (4) No detection of point mutations (5) Sensitive to small deletions, insertions, and mismatches (6) MLPA requires a CE analyzer which is a higher-cost option compared with slab gel electrophoresis for RFLP (7) Cannot distinguish silent carrier conditions (8) Cannot detect the presence of c.859G > A polymorphism in *SMN2* gene (9) Long work time (48 h)

**Table 3:** FDA-approved SMN-based DMTs for SMA [4,18,20,46,47,48,49].

Therapy	Mechanism of Action	Times and Method of Administration	Adverse Reactions	Age of Administration	Patients for Which It Is Intended
**Evrysdi** **(Risdiplam)**	Small molecule, splicing modifier of the *SMN2* gene	Orally, once a day (always at the same time) after eating	Pyrexia Skin rash Diarrhea	≥2 months	Patients with SMA 5q (mutations in the *SMN1* gene), 1 to 4 copies of the *SMN2* gene SMA 0 and SMA 4 patients excluded Not compatible with patients being treated with nusinersen or onasemnogene abeparvovec
**S** **pinraza** **(Nusinersen)**	Antisense oligonucleotide, splicing modifier of the *SMN2* gene	Intrathecal injection, on days 0, 14, 28, and 63; followed by a maintenance dose once every 4 months	Backache Heachache Nausea and vomiting	At birth	Patients with a genetically confirmed diagnosis of SMA 5q (mutations in the *SMN1* gene)
**Z** **olgensma** **(Onasemnogene abeparvovec** **)**	Non-replicating recombinant adeno-associated virus serotype 9 (AAV9)-based vector containing the cDNA of the human *SMN* gene	Intravenous injection by slow infusion over about an hour, once in a lifetime	Hepatotoxicity mainly. It often manifests as abnormal liver function, increased aminotransferases (AST, ALT), or rarely results in severe acute liver injury or acute liver failure, even with a fatal outcome	At birth	SMA 5q patients up to 13.5 kg: SMA 5q patients with a biallelic mutation in the *SMN1* gene with genetic or clinical diagnosis of SMA 1 Pre-symptomatic SMA 5q patients with a biallelic mutation in the *SMN1* gene and up to 3 copies of the *SMN2* gene

## Data Availability

Not applicable.
